# Interactions between Serum Adipokines and Osteocalcin in Older Patients with Hip Fracture

**DOI:** 10.1155/2012/684323

**Published:** 2012-02-22

**Authors:** Alexander Fisher, Wichat Srikusalanukul, Michael Davis, Paul Smith

**Affiliations:** ^1^Department of Geriatric Medicine, The Canberra Hospital, Canberra, P.O. Box 11, Woden, ACT 2606, Australia; ^2^Australian National University Medical School Canberra, Canberra, ACT 0200, Australia; ^3^Department of Orthopaedic Surgery, The Canberra Hospital, P.O. Box 11, Woden, ACT 2606, Australia

## Abstract

*Introduction*. Experiments on genetically modified animals have discovered a complex cross-regulation between adipokines (leptin, adiponectin) and osteocalcin. The relationships between these molecules in human osteoporosis are still unclear. We evaluated the hypothesis of a bidirectional link between adipokines and osteocalcin. *Materials and Methods*. In a cross-sectional study of 294 older patients with osteoporotic hip fracture, we estimated serum concentrations of leptin, adiponectin, resistin, osteocalcin, parameters of mineral metabolism, and renal function. *Results*. After adjustment for multiple potential confounders, serum osteocalcin concentration was inversely associated with resistin and positively with leptin, leptin/resistin ratio, and adiponectin/resistin ratio. In multivariate regression models, osteocalcin was an independent predictor of serum leptin, resistin, leptin/resistin, and adiponectin/resistin ratios. *Conclusions*. Our data support the bidirectional regulation between osteocalcin and adipokines, but contrary to the genetically modified animal models, in older subjects with osteoporotic hip fracture, serum osteocalcin is positively associated with leptin and inversely with resistin.

## 1. Introduction

Over the past two decades, it has been convincingly shown that adipose tissue is an active endocrine organ which produces a number of biologically active molecules named adipokines. More recently, the endocrine function of the skeleton and its important role in metabolic homeostasis has been revealed [1, 2]. Mainly through mouse genetic means by analysing loss-of-function models, the existence of a complex bilateral hormonal link (crosstalk) between bone and energy metabolism has been discovered [1–4]. According to the current paradigm, bone remodelling and energy metabolism are coregulated by adipocyte-derived hormones, leptin, and adiponectin, and the feedback loop between bone and energy metabolism is mediated by osteocalcin (OC), an osteoblast-specific protein. The biological importance of tight connections between adipose tissue and bone remodelling is further supported by the fact that adipocytes and osteoblasts are derived from a common mesenchymal progenitor cell [5], leptin and adiponectin are expressed in osteoblasts [6–8] and OC in human adipocytes [9]. A crosstalk between signalling pathway regulating adipocyte and osteoblast differentiation has also been recently described [10].

Results of experimental studies on reciprocal bone-energy metabolism relationships mediated by adipokines and OC are fairly consistent. However, clinical data on the association between circulating leptin and adiponectin levels and OC are controversial. Previous human studies that have evaluated the relationship between leptin and OC yielded conflicting results, showing either no correlation [11–16], positive [17], or negative correlation [18–21]. Similarly, some studies reported a positive association between serum adiponectin and OC [22–27], whereas other studies were not able to demonstrate a significant and independent relationship [16, 20, 28–30].

Emerging evidence has shown that resistin, a peptide hormone classified as an adipokine, although in humans it is mainly produced by mononuclear cells and macrophages, is important in regulating insulin resistance, diabetes, inflammatory processes, immunity, and bone metabolism [17, 31–34]. However, the interrelations between resistin and OC have not been characterised.

In the above-mentioned studies, such factors as serum calcium, phosphate, magnesium, 25-hydroxyvitamin D (25(OH)D), parathyroid hormone (PTH), renal status, and age, known to influence both bone metabolism and circulating adipokines, have rarely been measured and analysed. Lack of assessment of several adipokines simultaneously, difference in study populations and the dual nature of leptin's effect on the skeleton (central antiosteogenic [35] and peripheral osteogenic [5]) may also contribute to the inconsistency in human data.

There are only a few studies evaluating leptin [36, 37] and adiponectin [38] in patients with hip fracture (HF), but no research has been carried out showing the relationship between adipokines and OC in these patients. It remains to be determined whether the phenomenon of bidirectional adipokine-OC interaction is involved in human osteoporosis. Therefore, the aim of the present study was to asses in older patients with HF the association of leptin, adiponectin, and resistin, the three most widely investigated adipokines, with OC, and to examine whether OC is a significant and independent predictor of circulating adipokine levels. We also analysed the combined effect of adipokines using their ratios, since it has been suggested that metabolic functions of adipokines, especially of leptin and adiponectin, are complementary, and the leptin/adiponectin and adiponectin/resistin ratios are better clinical indicators [39–43].

## 2. Patients and Methods

### 2.1. Patients

A total of 294 consecutive older patients (≥60 years of age, mean age 82.1 ± 8.0 years) with low-trauma osteoporotic HF were included in this study. Data were obtained from a prospective electronic database on all adult patients with fracture of the upper femur admitted to the orthopaedic ward of The Canberra Hospital (Canberra, Australian Capital Territory, Australia), a university-affiliated tertiary care centre. Exclusion criteria were subtrochanteric and shaft fracture, age < 60 years, high trauma, and pathological HF due to primary or metastatic bone cancer, multiple myeloma, Paget disease, or primary hyperparathyroidism. Sociodemographic, anthropometric, clinical (HF type, co-morbidities, complications, medication use) and laboratory data were recorded.

Informed consent was obtained from all patients or their carers. The study has approval of the local Research Ethics Committee.

### 2.2. Laboratory Measurements

In all patients, antecubital venous blood samples were collected after overnight fast within 48 hours of arrival at the Emergency Department. Routine haematological and biochemical assessments were performed by standardised methods on autoanalysers at the day of collection. For assays of OC, leptin, adiponectin, and resistin serum samples were frozen in liquid nitrogen and stored at −70°C, subsequently thawed and analysed in a single batch using commercially available kits. Serum levels of OC were determined by electrochemiluminescence immunoassay (Elecsys 1010; Roche Diagnostics, IN, USA; analytical sensitivity 0.5 ng/mL, interassay coefficient of variation (CV) 2.1–3.1%, intraassay CV < 3%), leptin by enzyme-linked immunosorbent assay (ELISA) method (Diagnostic System Laboratories, Webster, TX, USA; sensitivity 0.05 ng/mL, interassay CV 3.4–5.5%, intraassay CV < 6%), total adiponectin and resistin by human ELISA kits (B-Bridge International, Mountain View, CA, USA; for adiponectin sensitivity 0.5 ng/mL, intraassay CV 3.2–7.3%, intraassay CV < 6%; for resistin sensitivity 0.03 ng/mL, interassay CV 4.5–7.2%, intraassay CV < 5%). All assays were performed with kits of the same lot number.

Serum levels of 25(OH)D were determined by a radioimmunoassay kit (Dia Sorin, Stillwater, MN, USA; sensitivity 0.7 pmol/L, interassay CV5.9–9.4%, intraassay CV < 11.5%), intact PTH by solid-phase two-site chemiluminescent enzyme-linked immunometricassay on a DPC Immulite 2000 analyzer (Diagnostic Products, Los Angeles, CA, USA; sensitivity 0.07 pmol/L, interassay CV 6.2–7.0%, intraassay CV < 6%). Serum calcium concentration was corrected for serum albumin. Glomerular filtration rate (eGFR) was estimated by the formula [44].

### 2.3. Statistical Analyses

All analyses were performed using Stata software (version 10; StataCorp, College Station, TX, USA). The summary statistics are presented as the mean ± standard deviation for continuous variables and as the number (percentages) for categorical variables. Continuous variables with a skewed distribution were logarithmically transformed before being used in correlation analyses. The relationships between variables were examined by Pearson's linear correlation test and multivariate logistic regression analyses. *P* < 0.05 (two-sided) was considered statistically significant. To assess the potential effect of multiple comparisons and the significance of multicollinearity phenomena in multivariate regression analyses, Bonferroni's and Sidak's corrections were used and the variance inflation factor was calculated.

## 3. Results

### 3.1. Patient Characteristics

The demographic and clinical characteristics of the study patients are shown in Table 1. There were 212 (72.1%) women and 82 (27.9%) men. Women were found to be slightly older than men (82.6 ± 7.7 versus 80.6 ± 8.3 years, *P* = 0.053). The HF was of cervical type in 52% and of trochanteric in 48% of patients. The mean (±SD) values of serum 25(OH)D and PTH were 37.2 ± 18.0 nmol/L and 6.9 ± 5.6 pmol/L, respectively. Vitamin D deficiency (25(OH)D < 50 nmol/L was found in 84.6% of females and 67.5% of males (*P* < 0.008) and secondary hyperparathyroidism (PTH > 6.8 pmol/L) in 39.4% and 25.3%, respectively (*P* = 0.028). The mean serum osteocalcin level was 17.2 ± 15.2 ng/mL. The serum osteocalcin levels were low (<14 ng/mL) in 53.3% of patients. The osteocalcin concentrations did not differ significantly with respect to gender or HF type. The main concentrations of serum leptin, adiponectin, and resistin were 18.4 ± 23.2 ng/mL, 17.5 ± 7.4 ng/mL, and 18.7 ± 10.5 ng/mL, respectively. Women had significantly higher mean serum concentrations of leptin (21.1 ± 24.3 versus 11.7 ± 18.6 ng/mL, *P* = 0.002), adiponectin (18.3 ± 7.1 versus 15.6 ± 7.6 ng/mL, *P* = 0.007), leptin/resistin ratio (1.6 ± 2.3 versus 0.8 ± 1, *P* = 0.006), and PTH (7.4 ± 6.1 versus 5.5 ± 3.5 pmol/L, *P* = 0.009), but lower levels of 25(OH)D (35.3 ± 17.6 versus 42.4 ± 18.2 nmol/L, *P* = 0.009), phosphate (0.89 ± 0.29 versus 1.07 ± 075 nmol/L, *P* = 0.003), magnesium (0.76 ± 0.13 versus 0.81 ± 0.12 nmol/L, *P* = 0.008), and eGFR (62.7 ± 22 versus 71.2 ± 26.4 mL/min./1.73m^2^, *P* = 0.006). Mean serum concentrations of resistin, leptin/adiponectin, and adiponectin/resistin ratios, osteocalcin, calcium (corrected for albumin), TSH, albumin, and haemoglobin in women and men did not differ.

 Malnutrition defined as serum leptin concentration <4 ng/mL in males and <6.5 ng/mL in females [45] was observed in 33.8% of patients (equal in both sexes). The malnourished group compared to the rest of the cohort was older (83.6 ± 7.8 versus 81.2 ± 8.0 years; *P* = 0.015) and as would be expected had higher serum levels of adiponectin (19.3 ± 6.6 versus 16.5 ± 7.5 ng/mL; *P* = 0.035) and lower levels of leptin (2.9 ± 1.3 versus 26.5 ± 25.0 ng/mL; *P* < 0.001), haemoglobin (121.3 ± 16.3 versus 126.3 ± 17.4 g/L; *P* = 0.021), leptin/adiponectin (0.17 versus 2.13; *P* < 0.001), and leptin/resistin (0.22 versus 1.94; *P* < 0.001) ratios; OC levels were also lower, however, the difference did not reach statistical significance (15.3 ± 9.7 versus 18.2 ± 17.3 ng/mL; *P* = 0.117).

Patients with cervical compared to trochanteric HF had higher serum levels of adiponectin (18.5 ± 7.3 versus 16.3 ± 7.3 ng/mL, *P* = 0.019) and resistin (20.1 ± 10.5 versus 16.9± ng/mL, *P* = 0.014), lower leptin/resistin ratio (1.1 ± 1.4 versus 1.7 ± 2.6, *P* = 0.025), and PTH concentrations (5.9 ± 3.6 versus 8.0 ± 6.9 pmol/L, *P* = 0.001), but did not differ significantly regarding other parameters.

### 3.2. Correlations of Adipokines with Serum Osteocalcin, Parameters of Mineral Metabolism, Renal Status, and Age

Pearson correlation analysis performed with log-transformed variables revealed that leptin correlated positively with osteocalcin (*r* = 0.123, *P* = 0.038), BMI (*r* = 0.210, *P* = 0.001), and haemoglobin (*r* = 0.188, *P* = 0.001) and inversely with adiponectin (*r* = −0.178, *P* = 0.005), phosphate (*r* = −0.161, *P* = 0.007), and age (*r* = −0.154, *P* = 0.009). Adiponectin correlated positively with PTH (*r* = 0.193, *P* = 0.002) and age (*r* = 0.251, *P* = 0.001) and negatively with BMI (*r* = −0.170, *P* = 0.005).

Resistin correlated positively with age (*r* = 0.156, *P* = 0.013) and negatively with serum magnesium (*r* = −0.198, *P* = 0.002) and eGFR (*r* = −0.126, *P* = 0.044). Serum osteocalcin correlated positively also with leptin/adiponectin ratio (*r* = 0.129, *P* = 0.041), leptin/resistin ratio (*r* = 0.166, *P* = 0.008), calcium (*r* = 0.169, *P* = 0.004), phosphate (*r* = 0.129, *P* = 0.003), magnesium (*r* = 0.124, *P* = 0.038), and age (*r* = 0.152, *P* = 0.010) and negatively with eGFR (*r* = −0.388, *P* = 0.001) and 25(OH)D (*r* = −0.127, *P* = 0.037).

### 3.3. Adipokines and Their Ratios as Independent Determinants of Serum Osteocalcin

Multiple regression analyses were performed to evaluate which individual adipokine or their ratios are independently associated with serum osteocalcin. As shown in Table 2, there was a significant positive correlation between serum log-leptin and log-osteocalcin before and after multiple adjustments. No relationship was found between log-transformed serum adiponectin and log-osteocalcin. Serum log-resistin was negatively and significantly associated with log-osteocalcin only after adjusting for age, sex, BMI, HF type, 25(OH)D, and PTH. This association remained significant after all further adjustments. In the final regression when all three adipokines were used in place of one, the independent determinants of serum osteocalcin were leptin (*P* = 0.040), resistin (*P* = 0.018), age (*P* = 0.018), and eGFR (*P* < 0.001). The model explained 39.5% of the variance in OC. Taking into account that type 2 diabetes mellitus (DM), hypertension, and other cardiovascular diseases which are common in the elderly population (Table 1) are known to be associated with dysregulation in adipokine metabolism, we further adjusted our models for these comorbidities (yes/no). These adjustments did not appreciably change the estimates for OC-leptin and OC-adiponectin associations. Neither hypertension (*per se*) nor any cardiovascular disease affected the OC-resistin relationship. However, addition of type 2 DM to the models with resistin made this association nonsignificant, indicating that higher resistin levels are incorporated in type 2 DM. Indeed, the patients with type 2 DM have significantly higher serum resistin concentrations (+29.2%) than the rest of the cohort (23.0 ± 11.2 versus 17.8 ± 10.0 ng/mL, *P* = 0.010).

Results of multivariate regression modelling testing the hypothesis that individual adipokine ratios are associated with serum osteocalcin are shown in Table 3. As it would be expected, there was a strong positive correlation between leptin/resistin ratio and serum log-osteocalcin, and it remained significant after all adjustments. There was also a positive association between leptin/adiponectin ratio and log-osteocalcin before and after adjusting for age, sex, BMI, HF type, and parameters of mineral metabolism, but this association lost significance when further adjusted for eGFR and haemoglobin as well as for comorbidities. In contrast, the adiponectin/resistin ratio was significantly associated with serum log-osteocalcin only in models near fully or fully adjusted.

Taken together, these results indicate that leptin and resistin are independent positive and negative, respectively, determinants of serum OC. Not surprisingly, the leptin/resistin ratio significantly and positively correlated with OC predicting 39.2% of the total variance in OC, and the adiponectin/resistin ratio could predict 37.4% of the variance. The partial associations between the leptin/adiponectin, adiponectin/resistin ratios and, OC confirm the independent effects of leptin and resistin on serum OC and show the influence of other factors related mainly to adiponectin.

### 3.4. Independent Factors Associated with Circulating Adipokines and Their Ratios

We next asked which factors are independent determinants of serum adipokine levels and their ratios. On multiple linear regression analyses and in keeping with previous results, serum OC was a significant predictor of both leptin and resistin, but not adiponectin (Table 4). Age was an independent determinant of each of the three adipokines. None of the adipokines was significantly and independently associated with 25(OH)D. Other parameters independently associated with circulating leptin were male sex, eGFR, adiponectin, (all three inversely), haemoglobin, and CVD (both positively). Serum resistin levels were independently associated with PTH (positively), trochanteric HF type, and serum magnesium (both inversely).

Independent predictors of the three adipokine ratios are shown in Table 5. OC was significantly independently associated with leptin/resistin and adiponectin/resistin ratios but not with the leptin/adiponectin ratio. Age, male sex (both negatively), trochanteric HF type, and haemoglobin (both positively) were the other parameters independently associated with the leptin/resistin ratio; the model explained 44.5% of variance in this ratio. OC was also the only independent predictor of the adiponectin/resistin ratio, but in fully adjusted model it accounted only 5.5% of variance.

## 4. Discussion

The main findings of this study of 294 consecutive unselected older patients with low-trauma HF are statistically significant correlations between leptin, resistin, and OC indicating complex interactions between adipocytes/monocytes/macrophages and osteoblasts. These have been demonstrated by simultaneous measurements of three major circulating adipokines (leptin, adiponectin, and resistin), their ratios, and serum OC. Multiple linear regression models adjusted for age, gender, BMI, HF type, key factors, or mineral metabolism (calcium, phosphate, magnesium, 25(OH)D, PTH), renal function, and haemoglobin showed that serum OC levels were significantly and positively associated with leptin and negatively with resistin concentrations, but not related to circulating adiponectin levels. Leptin, resistin (or leptin/resistin ratio), age, and eGFR were the only independent predictors of serum OC levels contributing to 39.5% of OC variance. On the other hand, OC was an independent determinant of serum leptin and resistin levels, as well as leptin/resistin and adiponectin/resistin ratios.

Although caution is needed when interpreting results of a cross-sectional study, our data may suggest the presence of adipokine-OC loops, specifically, leptin increases and resistin decreases OC secretion by osteoblasts, whereas circulating OC influences leptin (positive feedback loop) and resistin (negative feedback loop) production. These two counterbalancing circuits seem to be important components of a complex homeostatic framework. These results are in line with the current concept that efficient maintenance of metabolic homeostasis depends on interaction between adipose tissue/energy metabolism and skeleton [1–4], but the directions of some associations observed in this as in other clinical studies were opposite to that reported in experimental animals (genetically modified obese rodents).

Although adipokines, especially leptin and adiponectin, two pleiotropic hormones involved in regulation of a large variety of physiological processes, have been extensively studied in recent years, current data on their effects on bone and the adipokine-OC interactions in humans are controversial and the underlying mechanisms not fully understood.

It is now well acknowledged that the effect of leptin on bone is complex and includes different pathways: central inhibition of bone formation through the hypothalamus and brainstem involving *β* adrenergic, neuromedin U, neuropeptide Y, cocaine, and amphetamine-related transcript and serotoninergic systems [35, 46, 47] and direct peripheral (local) enhancement of osteoblastic cell differentiation, proliferation, and bone mineralization [5, 7]. Results from studies of the bone phenotype in animals with leptin deficiency (*ob/ob*) and leptin receptor deficiency (*dbldb*, *fa/fa*) are conflicting. While several groups concluded that leptin acts as a positive regulator of bone formation [48, 49], other suggested a negative (through the hypothalamus) effect on the skeleton [35, 47]. In humans, both positive and negative correlations between leptin and bone mineral density (BMD) [47], as well as with OC [11–21], have been reported.

Our finding that serum leptin is associated with OC is consistent with clinical observations that leptin positively correlated with OC [17] and BMD [15, 18, 50, 51] in different settings, whereas decrease of OC in bone predispose to HF [52]. Our results are also supported by a strong inverse association between serum leptin levels and nontraumatic fracture risk even in normal weight subjects [21], as well as the data that leptin enhances osteoblastogenesis *in vitro* [7, 48, 53], exerts a positive effect in fetal bone formation [54] and reduces bone loss in ovariectomized rats [55].

A positive association between leptin/adiponectin ratio and OC has been reported [17]. In our study, this association disappeared when eGFR, as an independent variable, was included in the regression model, and OC was not an independent predictor of the leptin/adiponectin ratio.

Taken together, it appears that leptin, which is involved in multiple endocrine pathways and exerts a wide spectrum of actions, may lead to opposite effects in different metabolic conditions. The negative effects of leptin on bone may predominant over the positive ones in obesity when leptin resistance occurs or when the serum leptin concentration rises above a certain threshold [50, 56]. In our study group, there were no obese persons and in 1/3 of patients the low serum leptin levels were indicative of malnutrition. In humans, energy deprivation and undernutrition with low leptinaemia are associated with low bone mass [57]. Our data suggests that in underweighted and normal weight persons, lower leptin levels are associated with decrease in OC, which in turn may further decrease leptin production. Leptin may have a therapeutic role in treating osteoporosis in undernourished patients.

Animal studies have indicated that OC regulates insulin metabolism through stimulating the expression of adiponectin in adipocytes [1–3]. However, the interrelation between OC and adiponectin remains unclear. Adiponectin and its receptors are expressed on osteoblasts, and adiponectin *in vitro *stimulates proliferation and differentiation of osteoblasts [6, 8, 58], and OC enhanced adiponectin expression in cultured adipocytes in a dose-dependent manner [2]. Experimental data *in vivo *(transgenic mice models) demonstrated all three a positive, negative or no effect of adiponectin on bone mass [58, 59]. Clinical studies reported more often a negative association [22, 60, 61], and also a positive [16] or no correlation [62]. Conflicting data on adiponectin-OC relationship [16, 20, 22–30] together with these discrepancies suggest that the effects may differ depending on other metabolic factors and clinical features. In the present study in concordance with other human studies [11, 17, 20, 30], no correlation was observed between serum adiponectin and OC. Serum adiponectin was not associated with BMD of proximal femur in patients with HF [38]. However, we found a positive relationship between adiponectin/resistin ratio and OC, suggesting that a shift in balance towards adiponectin may increase OC production by counteracting the action of resistin. Of note, this correlation was observed only when age, sex, BMI, HF type, parameters of mineral metabolism, and eGFR were included, as independent variables, in the regression analysis model. This indicates that serum adiponectin/resistin ratio is positively associated with OC levels in subjects with similar above-mentioned characteristics (e.g., when the influence of these variables is eliminated).

Our data demonstrate a reciprocal association between resistin and OC which has not been previously described. Resistin is expressed in mature human osteoblasts, and recombinant resistin increases osteoclastigenesis but only weakly affects differentiation of preosteoblasts into osteoblasts [63]. The few clinical studies of resistin-bone relationship provided conflicting data [17, 30, 61, 64]. Our results are in line with observations that resistin inversely correlates with BMD in the hip [64], lumbar spine [30], and radius [65]. Remarkably, the resistin–OC association in our study became significant only after controlling for 25(OH)D and PTH and remained significant after further adjustments for all other covariates except type 2 DM (Table 2), suggesting that this relationship can be masked by parameters of mineral metabolism. Indeed, our data showed that PTH and magnesium were independent determinants of circulating resistin but not leptin (Table 5) providing a potential explanation for the “masked” effect.

Of note, after adjusting for type 2 DM, the association between OC and resistin became nonsignificant, suggesting that common signalling and metabolic pathways for OC and resistin contribute to DM. In line with other studies [66–69], we found that serum resistin levels in patients with type 2 DM were significantly higher compared to the rest of the cohort. These observations are consistent with growing evidence that resistin may be a potential mediator of DM [70–73] acting, at least partially, through OC.

The relationships between adipokine ratios and OC have not been systematically examined previously. In this study, after full adjustment, a significant interrelation between the leptin/resistin ratio and OC and between adiponectin/resistin ratio and OC was found. However, analyses of these ratios did not yield additional information compared to either leptin or resistin measurements.

It should be pointed out that multiple regression analysis explained only 39.5% of the variance in serum OC, indicating that factors other than leptin and resistin significantly influenced the level of OC. We found that both eGFR and age are independently associated with serum OC as well as leptin levels, and age is an independent determinant of resistinaemia. Consistent with other studies, ours showed that deterioration of kidney function was associated with higher OC [22] and leptin [74] levels. There is an age-dependent decrease in proliferation and differentiation of human osteoblasts, and the highest OC levels have been reported during adolescence [75]. However, in adults, no correlation between OC and age was found in some studies [11], while other described decreased OC levels with age [76]. In about half (53.3%) of our patients, the serum OC concentration was low (lower than the low limit of the reference range) but it was significantly and positively associated with age. It is reasonable to consider that renal dysfunction may at least partially explain this association, as we observed a strong negative correlation between age and eGFR (*r* = −0.313, *P* = 0.001) and eGFR was markedly decreased (<60 mL/min./1.73 m^2^) in 42.6% of our patients.

**Figure 1:**
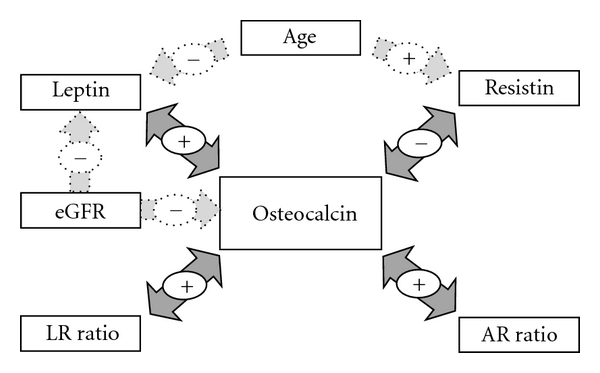
Schematic representation of independent significant associations between serum osteocalcin, adipokines, their ratios, renal status, and age in older patient with hip fracture. LR, leptin/resistin ratio; AR, adiponectin/resistin ratio, eGFR, estimated glomerular filtration rate. Bidirectional interactions exist between leptin and osteocalcin (positive), between resistin and osteocalcin (negative) and as a consequence between leptin/resistin ratio and osteocalcin and between adiponectin/resistin ratio and osteocalcin (both positive). Age is an independent determinant of circulating levels of resistin (positive association) and leptin (negative associations), whereas renal function (eGFR) is inversely associated with osteocalcin and leptin.

The complex interplay of many metabolic, renal, and age-related factors may account for some of the discrepancies in the literature in regard to adipokine-OC interactions. It is possible that various combinations of these factors are causing distinct positive or negative effects. Further complicating the matter, adiponectin and resistin (as well as leptin) have been shown to be neuroendocrine hormones acting directly on the brain [77–81], but in contrast to leptin, the centrally mediated effects of adiponectin and resistin on osteoblast functions are unknown. Moreover, receptors for resistin and OC still remain unidentified. To develop an integrated understanding of adipokine-bone interaction, a lot more work is needed to be done. There is a growing body of evidence demonstrating that in osteoporosis impaired bone metabolism, including OC production and secretion, does not exist in isolation. It reflects the alterations in a highly complex homeostatic system. Our data indicate the existence of bidirectional leptin-OC (positive) and resistin-OC (negative) relationships as a part of a complex energy metabolism-bone network in older patients with HF.  Figure 1 represents the complex interactions of OC with adipokines depicting independent significant associations between OC, circulating adipokines, their ratios, age, and renal status in older patients with HF. Further examination of the role of these interactions in osteoporotic fractures and metabolic disorders was warranted.

In regard to the differences in OC-adipokine interactions observed in humans and rodents, it should be noted that, firstly, the *Esp *gene, specifically studied in knockout mouse models, is a pseudogene in humans [82]. No functional *Esp* gene has been identified in humans, although a close homologue of Esp is expressed in human osteoblasts [83]. Secondly, in genetically modified rodents, changes in OC and adipokine levels are much larger than in clinical observations. Thirdly, the compensatory mechanisms caused by genetic manipulations are not presented in humans. Fourthly, the effects of ageing and comorbidities have not been addressed in animal studies. 

The notable strength of this study is simultaneous assessment of three circulating adipokines and OC in the same cohort and adjustment for a wide range of confounding factors, the major limitations of previous studies. However, multiple comparisons in multivariate regression analysis may potentiate multicollinearity. After Bonferroni and Sidak adjustments, all determinants preserved statistical significance, and in all our models (Tables 2–5), the variance inflation factor was between 1.21 and 1.27 indicating that the amount of multicollinearity was not significant. The main limitation of our study is its cross-sectional design which precludes conclusions regarding causality. Another potential limitation of this study is that only total OC and total adiponectin have been measured. The animal and *in vitro* studies showed that uncorboxylated OC exerts an effect on glucose homeostasis and energy metabolism [1]. However, other studies reported that both carboxylated and uncarboxylated forms of OC and total OC are associated with glucose metabolism and insulin resistance [11, 23, 24, 76]. Similarly, some studies concluded that high-molecular weight (HMW) adiponectin is a better predictor of insulin resistance and metabolic syndrome, while other studies did not find a significant difference between HMW and total adiponectin in this regard. We cannot exclude the possibility that measurements of specific forms of OC and adiponectin may provide different results. Finally, our study population represents almost exclusively elderly Caucasians, and the results may have limited applicability to other age and ethnic groups.

In conclusion, in older patients with HF, leptin is directly and resistin inversely associated with circulating OC, and OC is a significant independent determinant of both serum leptin (positive) and resistin (negative) concentrations. These suggest bidirectional interactions (crosstalk) between leptin, resistin and OC as a part of a complex homeostatic system regulating bone and energy metabolism. Our data do not support an independent link between adiponectin and OC in these patients. Further studies should be performed to evaluate the role of leptin-OC and resistin-OC axes in osteoporotic fractures and comorbid conditions such as cardio- and cerebrovascular diseases, diabetes, dementia, malnutrition, all of which are common in the elderly and have been shown to be associated with alterations in serum adipokine and OC levels.

## Figures and Tables

**Table 1:** Demographic and clinical characteristics of the study patients with hip fracture (*n* = 294).

Characteristic	

Age, years (mean ± SD)	82.2 ± 7.9
Females, %	72.1

Comorbidities:	

Hypertension, %	54.0
CAD, %	21.2
Previous myocardial infarction, %	5.3
Atrial fibrillation, %	13.2
History of stroke, %	14.3
TIA, %	7.4
Type 2 DM, %	18.8
Dementia, %	27.8
Parkinson's disease	4.4
CKD stage ≥ 3	42.6
COPD	11.8
Malnutrition	33.8

CAD, coronary artery disease; TIA, transient ischaemic attack; DM, diabetes mellitus; CKD, chronic kidney disease; COPD, chronic obstructive pulmonary disease.

**Table 2:** Associations of serum leptin, adiponectin, and resistin with serum osteocalcin as dependent variable in linear regression models.

Adjustments	Leptin		Adiponectin		Resistin	
	*β*	*P*	*β*	*P*	*β*	*P*
Unadjusted	0.071	**0.038**	−0.015	0.859	−0.064	0.372
^+^Age, sex, BMI	0.093	**0.008**	−0.087	0.329	−0.099	0.167
^+^HF type	0.093	**0.009**	−0.084	0.350	−0.097	0.182
^+^25(OH)D	0.081	**0.023**	−0.126	.0162	−0.138	0.055
^+^PTH	0.080	**0.024**	−0.138	0.129	−0.169	**0.022**
^+^Ca, Mg, PO_4_	0.079	**0.027**	−0.123	0.072	−0.178	**0.019**
^+^eGFR	0.055	0.105	−0.073	0.406	−0.162	**0.026**
^+^Haemoglobin	0.076	**0.046**	−0.075	0.394	−0.162	**0.026**
^+^Two other adipokines	0.077	**0.040**	−0.051	0.567	−0.173	**0.018**
^+^Type 2 DM	0.090	**0.018**	−0.053	0.551	−0.119	0.126
^+^Hypertension*	0.078	**0.046**	−0.055	0.544	−0.173	**0.021**
^+^CVD (any)*	0.090	**0.023**	−0.059	0.575	−0.156	**0.037**
^+^CVD (any)	0.099	**0.014**	−0.061	0.507	−0.104	0.191

Leptin, adiponectin, resistin, and osteocalcin were included in models as logarithmically transformed variables; ^+^indicates adjustments for all covariates in the above model; *indicates adjustments for all covariates in the above model except type 2 DM; *β* standard regression coefficient; *P* probability value; DM, diabetes mellitus; CVD, cardiovascular disease; BMI, body mass index; HF, hip fracture; 25(OH)D, 25 hydroxy vitamin D; PTH, parathyroid hormone; Ca, calcium; Mg, magnesium; PO_4_, phosphate; eGFR, estimated glomerular filtration rate.

**Table 3:** Associations of serum adipokine ratios with serum osteocalcin as dependent variable in linear regression models.

Adjustments	Leptin/Adiponectin ratio		Leptin/Resistin ratio		Adiponectin/Resistin ratio	
	*β*	*P*	*β*	*P*	*β*	*P *
Unadjusted	0.037	**0.041**	0.055	**0.008**	0.047	0.098
^+^Age, sex, BMI	0.048	**0.007**	0.079	**0.000**	0.044	0.114
^+^HF type	0.048	**0.008**	0.079	**0.000**	0.044	0.115
^+^25(OH)D	0.039	**0.028**	0.071	**0.001**	0.045	0.095
^+^PTH	0.042	**0.019**	0.075	**0.000**	0.048	0.076
^+^Ca, Mg, PO_4_	0.041	**0.023**	0.068	**0.002**	0.048	0.084
^+^eGFR	0.031	0.074	0.057	**0.007**	0.052	**0.042**
^+^Haemoglobin	0.033	0.064	0.061	**0.005**	0.052	**0.043**
^+^type 2 DM	0.034	0.055	0.058	**0.006**	0.035	0.426
^+^Hypertension*	0.034	0.060	0.061	**0.005**	0.053	**0.044**
^+^CVD (any)*	0.034	0.062	0.060	**0.005**	0.051	**0.050**
^+^CVD (any)	0.034	0.057	0.057	**0.009**	0.038	0.453

Osteocalcin was included in the models as a logarithmically transformed variable. ^+^indicates adjustments for all covariates in the above model; *Indicates adjustments for all covariates in the above model except type 2 DM; *β* standard regression coefficient; *P* probability value; DM, diabetes mellitus; CVD, cardiovascular disease; BMI, body mass index; HF, hip fracture; 25(OH)D, 25 hydroxy vitamin D; PTH, parathyroid hormone; Ca, calcium; Mg, magnesium; PO_4_, phosphate; eGFR, estimated glomerular filtration rate.

**Table 4:** Independent factors associated with serum adipokine levels in older patients with hip fracture (multivariate linear regression models).

	Leptin		Adiponectin		Resistin	
	*β*	*P*	*β*	*P*	*β*	*P*
Osteocalcin	0.297	**0.014**	−0.036	0.507	−0.133	**0.037**
Age	−0.037	**0.001**	0.015	**0.002**	0.011	**0.050**
Sex (m)	−0.598	**0.001**	−0.184	**0.020**	0.074	0.437
HF type	0.215	0.163	−0.209	**0.002**	−0.208	**0.010**
25(OH)D	−0.006	0.129	0.003	0.155	0.002	0.417
PTH	0.001	0.949	0.020	**0.003**	0.021	**0.006**
Ca	−1.056	0.090	−0.216	0.436	0.617	0.059
PO_4_	−0.273	0.057	0.049	0.439	−0.059	0.443
Mg	0.323	0.581	−0.307	0.236	−0.924	**0.003**
eGFR	−0.009	**0.024**	0.0011	0.430	−0.001	0.829
Adiponectin	−0.331	**0.036**	—	—	−0.045	0.621
Leptin	—	—	−0.065	**0.036**	0.003	0.945
Resistin	−0.063	0.649	−0.018	0.769	—	—
Haemoglobin	0.019	**0.036**	−0.002	0.429	−0.001	0.767
Type 2 DM	0.232	0.269	−0.120	0.197	0.117	0.261
CVD (any)	0.449	**0.004**	−0.016	0.827	0.103	0.224

Leptin, adiponectin, resistin and osteocalcin were included in models as logarithmically transformed variables; *β* standard regression coefficient; *P* probability value; BMI, body mass index; HF, hip fracture; 25(OH)D, 25 hydroxy vitamin D; PTH, parathyroid hormone; Ca, calcium; Mg, magnesium; PO_4_, phosphate; eGFR, estimated glomerular filtration rate; DM, diabetes mellitus; CVD, cardiovascular disease.

**Table 5:** Independent factors associated with serum adipokine ratios in older patients with hip fracture (multivariate linear regression models).

	Leptin/Adiponectin ratio		Leptin/Resistin ratio		Adiponectin/Resistin ratio	
	*β*	*P*	*β*	*P*	*β*	*P*
Osteocalcin	0.511	0.057	0.577	**0.009**	0.354	**0.044**
Age	−0.082	**0.000**	−0.094	**0.000**	0.010	0.521
Sex (m)	−0.763	0.051	−0.107	**0.001**	−0.432	0.096
HF type	0.898	**0.007**	0.782	**0.004**	−0.028	0.897
25(OH)D	0.024	**0.013**	−0.010	0.175	0.001	0.910
PTH	−0.082	**0.018**	−0.047	0.095	−0.020	0.357
Ca	−0.205	0.882	−0.329	0.771	−1.086	0.220
PO_4_	−0.478	0.136	−0.275	0.293	0.121	0.570
Mg	2.077	0.108	2.011	0.058	1.270	0.140
eGFR	−0.015	0.095	−0.011	0.127	0.007	0.208
Haemoglobin	0.026	**0.008**	0.026	**0.001**	−0.002	0.728
Type 2 DM	−0.005	0.991	−0.110	0.775	−0.290	0.127
CVD (any)	0.232	0.508	0.141	0.622	−0.169	0.234

Leptin, adiponectin, resistin and osteocalcin were included in models as logarithmically transformed variables; *β* standard regression coefficient; *P* probability value; BMI, body mass index; HF, hip fracture; 25(OH)D, 25 hydroxy vitamin D; PTH, parathyroid hormone; Ca, calcium; Mg, magnesium; PO_4_, phosphate; eGFR, estimated glomerular filtration rate; DM, diabetes mellitus; CVD, cardiovascular disease.
